# Atraumatic Splenic Rupture in a SARS-CoV-2 Patient: Case Report and Review of Literature

**DOI:** 10.1155/2021/5553619

**Published:** 2021-06-04

**Authors:** Marcello Agus, Maria Elena Ferrara, Paola Bianco, Cristina Manieli, Paolo Mura, Raffaele Sechi, Matteo Runfola, Fabrizio Polo, Nicola Cillara

**Affiliations:** ^1^S.S.D. Chirurgia d'Urgenza, P.O. San Michele, ARNAS “G Brotzu, ”Cagliari, Italy; ^2^U.O. Medicina di Accettazione e d'Urgenza, P.O. San Michele, ARNAS “G Brotzu, ”Cagliari, Italy; ^3^U.O. Anatomia Patologica, P.O. San Michele, ARNAS “G Brotzu, ”Cagliari, Italy; ^4^S.O.C. Chirurgia Generale, P.O. Santissima Trinità, ASL Cagliari, Italy

## Abstract

Splenic rupture in the absence of trauma or previously diagnosed disease is rare. Due to the delay of diagnosis and treatment, this is a potentially life-threatening condition. We report a case of atraumatic splenic rupture in a SARS-CoV-2 patient. This report is of particular interest as it first identifies SARS-CoV-2 infection as a possible cause of spontaneous rupture of the spleen. A 46-year-old Caucasian woman presented at the emergency department pale and sweaty, complaining of syncopal episodes, tachycardia, hypotension, diarrhea, intense abdominal pain, diffuse arthromyalgia, and fever from the day before. RT-PCR was positive for SARS-CoV-2 infection. CT scan demonstrated extensive hemoperitoneum due to rupture of the splenic capsule. The patient required an emergency open splenectomy because of an unresponsive hemorrhagic shock. At the end of the surgery, the patient was relocated to a COVID-19 dedicated facility. COVID-19 is a new disease of which all manifestations are not yet known. Inpatients affected by SARS-CoV-2 infection with abdominal pain and spontaneous splenic rupture should be considered to avoid a delayed diagnosis.

## 1. Background

Severe acute respiratory syndrome (SARS) coronavirus- (CoV-) 2 (SARS-CoV-2), the etiological agent that causes coronavirus disease 2019 (COVID-19), which was declared a pandemic by the World Health Organization (WHO) in March 2020, presents principally as a lower respiratory tract infection, but the multisystemic nature of the disease is evident in severe cases. Indeed, a broad spectrum of symptoms associated with COVID-19 has been identified. These range from asymptomatic disease to mild and moderate symptoms and severe symptoms associated with critical illness resulting in acute respiratory distress syndrome, respiratory failure or multiorgan dysfunction, and/or death [1, 2].

The tropism of coronaviruses for the spleen has already been shown in patients infected by SARS-CoV [3, 4].

The atraumatic rupture of the spleen is widely documented in the literature and is associated with a previous pathological finding in 93% of cases [5]. Infections cause about a quarter of atraumatic splenic ruptures and are commonly related to the Epstein-Barr virus and malaria [6]. Emergency splenectomy is generally indicated in the management of hemodynamically unstable patients with splenic rupture, a reason for which an accurate and rapid diagnosis of splenic rupture is crucial in preventing significant morbidity and mortality. The case we present here shows the occurrence of an atraumatic splenic rupture secondary to SARS-CoV-2 infection.

## 2. Case Presentation

A 46-year-old Caucasian woman presented to the emergency department pale and clammy, complaining from the day before of a syncopal episode, hypotension, diarrhea, and left shoulder tip pain which in the immediate hours before hospitalization spread to the whole abdomen. The patient also complained from the day before of widespread arthromyalgia and slight fever. No history of trauma was reported.

Her past surgical history included a laparoscopic sleeve gastrectomy performed for obesity (pretreatment BMI 37.5) about a year earlier. At presentation, the patient had a BMI of 25.3.

At the preliminary examination, she was alert and oriented, tachycardic at 100 beats per minute, and normotensive and had a temperature of 35.8°C. She was eupnoeic with an oxygen saturation of 99% on air. Examination of the abdomen revealed generalized guarding at palpation, worst in the left hypochondrium. The hemoglobin level at arrival was 7.7 g/dl (12-17.5), and she received a transfusion of 1 unit of red blood cells. Diffuse free fluid in the abdomen was seen on the patient's Focused Assessment with Sonography for Trauma (FAST) ultrasound. Reverse transcriptase-polymerase chain reaction (RT-PCR) was positive for SARS-CoV-2 infection.

The routine admission blood test revealed a liver function test partially deranged with normal bilirubin, alkaline phosphatase, and gamma glutamic transpeptidase, but abnormal alanine aminotransferase 69 IU/L (0-45) and aspartate aminotransferase 130 IU/L (0-35). At presentation, the coagulation values were PT INR 1.12 (0.8-1.2), APTT sec. 25 (17-35), and APTT ratio: 0.9 (0.8-1.2). The platelets were 168 × 10 · *e*3/*μ*L (130-400).

The rest of her routine biochemical investigation was unremarkable, and neither portal hypertension nor liver steatosis was documented either clinically or macroscopically.

**Figure 1:**
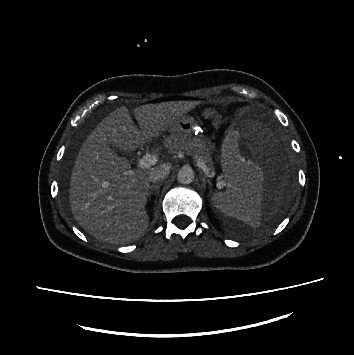
CT abdomen (axial) demonstrating subcapsular hematoma.

**Figure 2:**
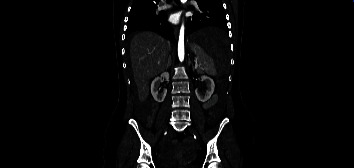
CT abdomen (coronal) demonstrating hemoperitoneum.

Chest and abdominal CT scan revealed parenchymal thickening areas associated with frosted glass opacity halo in both lower lobes of inflammatory significance (lung score 2), voluminous splenic subcapsular hematoma with a maximum thickness of 57 mm, and abundant hemoperitoneum (Figures 1 and 2).

During this investigation, the patient became increasingly unstable with blood pressure 75/45 mmHg and cardiac frequency > 100 pulse rate per minute and mildly anxious, which is why the patient, in accordance with the literature, had an emergency (rather than laparoscopic) laparotomy within 2 hours of presentation. This revealed 2 L of free blood in the peritoneal cavity and a bulky blood clot attached to the lower pole of the broken spleen [7].

Choice in the surgical approach was not conditioned by COVID-19 infection, even though previous research has shown that laparoscopy may lead to aerosolization of blood-borne viruses. Currently, there is no evidence to indicate that this effect is seen with COVID-19, nor that it would be limited to minimally invasive surgery procedures [8].

On the first postoperative day, the patient was transferred to a COVID-19 surgical unit where she needed antibiotic treatment with azithromycin and oxygen low-flow therapy for a week.

Both acute-phase and convalescent serum analyses provided no evidence of other acute viral infections beyond SARS-CoV-2. The postoperative course was complicated by a wound infection, and the patient was discharged on day 20 after therapy.

**Figure 3:**
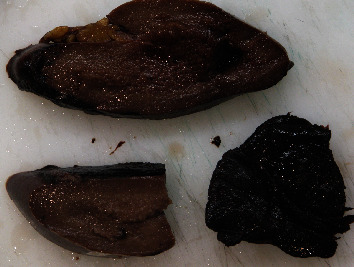
Macroscopy multiple hematomas are present underneath the capsule.

**Figure 4:**
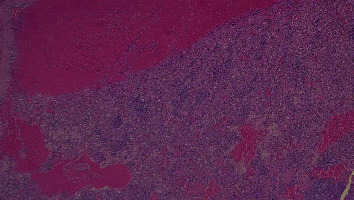
10x EE: subcapsular hematoma (upper) and expansion of red pulp of the spleen that appears congested with multiple extravasated erythrocytes.

**Figure 5:**
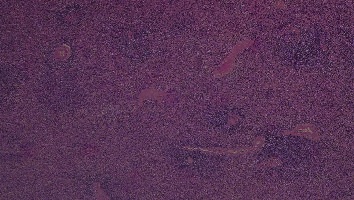
4x EE: expansion of red pulp and normal lymphoid follicles in the white pulp.

**Figure 6:**
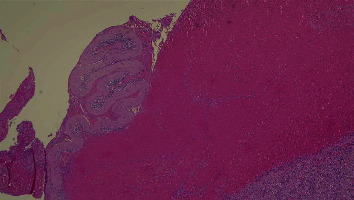
4x E-E: the capsule of the spleen is torn apart and a voluminous hematoma is compressing the spleen's parenchyma beyond it.

At macroscopy, the pathology report demonstrated that the spleen had a normal size with a weight of 182 g, the capsule was torn on multiple sides, and several subcapsular hemorrhages and hematomas were documented with a variable range from 4.5 cm to 2 cm (Figure 3). The larger one was on the anterior surface of the spleen and compressed the parenchyma behind and probably led to the spleen capsule rupture. At microscopy, the white pulp showed normal compartmentalization of B and T lymphocytes, demonstrated also by immunohistochemistry (CD3 and CD20), and the red pulp showed sinuses normal in size but increased in number, with a slight decrease in chordal tissue and capillaries (Figures 4–6). We also performed a novel immunohistochemistry marker SARS-CoV/SARS-CoV-2 (COVID-19) spike antibody in the spleen of our patient to eventually demonstrate SARS-CoV-2 presence in the tissue.

The case shows the effective use of the multidisciplinary hospital team to diagnose and manage what was a potentially life-threatening event.

## 3. Discussion

Coronavirus disease 2019 (COVID-19), first identified in December 2019 in Wuhan, China, is an infectious disease caused by severe acute respiratory syndrome coronavirus 2 (SARS-CoV-2).

Clinical manifestations and disease course of COVID-19 are extremely variable in different individuals, as it depends on the balance between SARS-CoV-2 virulence and the host's characteristics.

Distinctive symptoms of COVID-19 are cough, fever, dyspnea, myalgia, and fatigue, but patients may also experience gastrointestinal manifestations and related multiorgan complications [9].

During the SARS-CoV-2 pandemic, it has been seen that the disease appears to have a strong thrombotic tendency due to thromboinflammation, probably driven by distinct but as yet uncertain processes. These mechanisms may predispose patients to arterial and venous thrombosis, although risk estimates for these complications have not yet been properly defined [1].

Autopsy examinations have demonstrated microcirculation damage in patients with COVID-19 [10], and recent studies suggest that hemostatic abnormalities, including disseminated intravascular coagulation (DIC), occur in such patients [11]. These hemostatic changes indicate some forms of coagulopathy that may predispose to thrombotic events, although the cause is uncertain [12]. Whether the hemostatic changes are a specific effect of SARS-CoV-2 or whether they are a consequence of a cytokine storm that precipitates the onset of systemic inflammatory response syndrome (SIRS), as observed in other viral diseases, is not completely understood [13, 14]. More data are required on how COVID-19 and thrombotic disease interact.

As demonstrated in the case described, there is a wide range of clinical signs and symptoms associated with SARS-CoV-2, but the potential for life-threatening sequelae exists.

To date, cases of splenic infarction associated with SARS-CoV-2 infection are rare in the literature. However, arteriolar thrombosis and splenic infarction were observed in one patient in a study that evaluated splenic pathological changes identified on autopsy in 10 cases of COVID-19 [14].

Causes of splenic rupture can be divided into three categories: traumatic, nontraumatic, and spontaneous. Spontaneous splenic rupture as defined by Orloff and Peskin in 1958 is extremely rare [15].

Nontraumatic rupture of the spleen instead, although rare, is a complication of a series of different diseases.

Renzulli identifies six etiological groups among which infections represent about 27% of cases. Mononucleosis and malaria are considered to be the most common infectious causes. However, other causes of rupture secondary to infection, such as cytomegalovirus, HIV, and dengue fever [16], are documented.

We performed literature research using PubMed (search terms “splenic rupture” or “splenectomy” and “SARS-CoV2” or “COVID-19”). We found three recently described cases of splenic rupture due to SARS-CoV-2 infection. Of these, two required splenectomy [17] and the other underwent splenic artery embolization [18].

Karki et al. instead described the case of a COVID-19 patient with a hemoperitoneum due to infarct laceration of the splenic capsule which resolved spontaneously [19].

**Figure 7:**
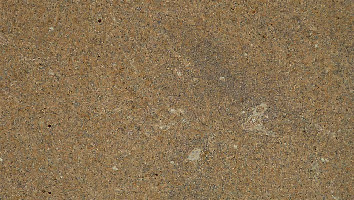
10x: IHC SARS-CoV/SARS-CoV-2 (COVID-19) spike antibody resulted negative.

**Figure 8:**
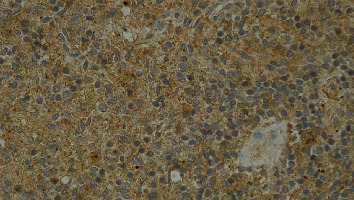
40x: IHC SARS-CoV/SARS-CoV-2 (COVID-19) spike antibody resulted negative.

In our case, immunohistochemistry was performed to identify the SARS-CoV-2 viral spike protein using SARS-CoV/SARS-CoV-2 (COVID-19) spike antibody (Genetex clone 1A9 at 1 : 75 dilution with 20 min antigen retrieval at pH 9.0 on Leica Bond III automated instrument) in the spleen of our patient, and it resulted negative (Figures 7 and 8). There was no sign of an inflammatory process, and an alternative explanation may be described as endothelial dysfunction and dysregulation even in the absence of morphological features.

It may be that in this patient, the rupture was the result of a progressive subcapsular hematoma that subsequently ruptured the capsule and caused hemoperitoneum (Figures 1 and 2). Some recent studies analyzed the interaction between endothelium infections and dysregulation by SARS-CoV-2 [20]. SARS-CoV-2 may thus cause endothelial dysfunction either directly through endothelial cell infection or indirectly through the infection of other susceptible cell types, which cause hyperinflammation and aberrant antiviral responses [21, 22]. Endothelial dysregulation accordingly results in vasoconstriction, vascular leakage, thrombosis, hyperinflammation, and dysregulation of the antiviral immune response [23].

Another recent study analyzed the presence of SARS-CoV-2 in the spleen and lymph nodes and found that in contrast to normal healthy controls, the lymph follicles and paracortical areas in virus-infected tissues are not identifiable, with necrotic and apoptotic lymphocytes being widely distributed, causing a significant reduction in total lymphocytes, including cells in T and B zones. Moreover, the spleen was congested and hemorrhagic with atrophy of the spleen's corpuscles and fibrous tissue hyperplasia in the splenic sinus.

For the authors, these results demonstrate that SARS-CoV-2 causes severe damage in the human spleen and lymph nodes through both direct and indirect mechanisms [24].

## 4. Conclusion

The clinical spectrum of SARS-CoV-2 infection ranges from asymptomatic infection to critical illness.

It is important to know the importance of splenic infarction as a thrombotic complication of COVID-19, also considering the fact that in the pre-COVID-19 era the diagnosis for this case would have been “spontaneous rupture of spleen,” since other diagnostic hypotheses could not be formulated.

It is extremely important to identify complications associated with this disease. Although thrombotic events are one of the main complications of SARS-CoV-2 infection, reports and imaging findings of splenic infarction are rare in the literature. The incidence of splenic infarctions is probably underestimated since abdominal imaging is not routinely performed, and they are often incidental findings from chest CT scans that extend to the abdomen.

We wish to underline that SARS-CoV-2 infection is a systemic disease that can affect different organs and tissues; albeit the pathogenesis of splenic rupture in COVID-19 patients is not fully understood, limited also by paucity of cases, we hypothesize that in our patient congestion of spleen parenchyma, due to endothelial dysregulation by SARS-CoV-2 infections, may lead to increasing vasculature permeability and leaking in microvasculature structures with spontaneous formation of subcapsular hematoma.

Nontraumatic splenic rupture should be considered promptly in patients with abdominal pain suffering from SARS-CoV-2 infection, and this new pathogen should be considered a possible cause of splenic rupture along with other more common infectious precedents, neoplasms, and hematological diseases.
